# Influence of prior information on pain involves biased perceptual decision-making

**DOI:** 10.1016/j.cub.2014.06.022

**Published:** 2014-08-04

**Authors:** Katja Wiech, Joachim Vandekerckhove, Jonas Zaman, Francis Tuerlinckx, Johan W.S. Vlaeyen, Irene Tracey

**Affiliations:** 1 Oxford Centre for Functional Magnetic Resonance Imaging of the Brain, Univ. of Oxford, JR Hospital, Oxford OX3 9DU, UK; 2 Nuffield Dept. of Clinical Neurosciences, Univ. of Oxford, JR Hospital, Oxford OX3 9DU, UK; 3 Dept. of Cognitive Sciences, 2324 SBSG, UC Irvine, CA 92697-5100, USA; 4 Faculty of Psychology and Educational Sciences, Univ. of Leuven, Tiensestraat 102, 3000 Leuven, Belgium; 5 Dept. Clinical Psychological Science, Maastricht University, P.O. Box 616, 6200 MD Maastricht, Netherlands

## Abstract

Prior information about features of a stimulus is a strong modulator of perception. For instance, the prospect of more intense pain leads to an increased perception of pain, whereas the expectation of analgesia reduces pain, as shown in placebo analgesia and expectancy modulations during drug administration [1]. This influence is commonly assumed to be rooted in altered sensory processing and expectancy-related modulations in the spinal cord [2], are often taken as evidence for this notion. Contemporary models of perception, however, suggest that prior information can also modulate perception by biasing perceptual decision-making — the inferential process underlying perception in which prior information is used to interpret sensory information. In this type of bias, the information is already present in the system before the stimulus is observed [3]. Computational models can distinguish between changes in sensory processing and altered decision-making as they result in different response times for incorrect choices in a perceptual decision-making task (Figure S1A,B) [4]. Using a drift-diffusion model, we investigated the influence of both processes in two independent experiments. The results of both experiments strongly suggest that these changes in pain perception are predominantly based on altered perceptual decision-making.

## Teaser

The expectation of pain or analgesia can increase or decrease, respectively, the perceived intensity of the pain. Wiech et al. show that this effect is predominantly due to altered perceptual decision-making rather than changes in sensory processing, as often assumed.

## Main Text

Thirty-four right-handed healthy volunteers (23 female; mean age 23.4 years) took part in the study in Experiment 1. In a probabilistic cueing paradigm, participants were presented with one of two visual cues in each trial. Cue 1 signaled the subsequent application of a high intensity noxious electrical stimulus with a probability of 80% and of a low intensity stimulus with a probability of 20%. Cue 2 signaled an equal probability (i.e. 50%) for both high and low intensity stimuli. To test whether the results of Experiment 1 were specific for prior information about high intensity pain, we conducted a second experiment (N = 22; 11 female; mean age 26 years) with an additional condition in which a third cue signaled a prior probability of 20% for high intensity pain and an 80% probability for low intensity pain.

**Figure 1:**
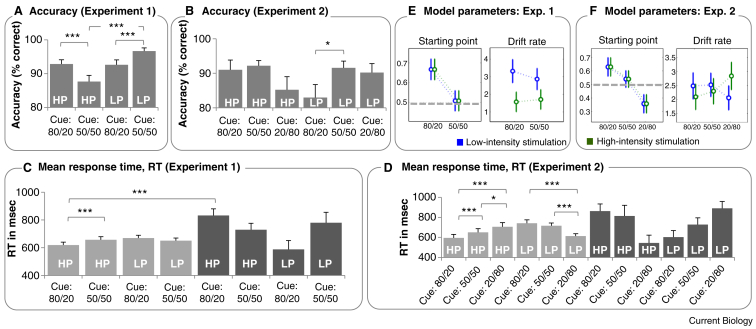
Biased sensory processing or altered perceptual decision-making? Mean decision accuracies for the four experimental conditions in Experiment 1 (A) and the six conditions in Experiment 2 (B) (HP, high intensity pain; LP, low intensity pain). (C,D) Mean response times for correct responses (light grey) and incorrect responses (dark grey; HP, high intensity pain; LP, low intensity pain). (E,F) The group average of the modelling parameters starting point (left) and drift rate (right) in Experiment 1 (E) and Experiment 2 (F). The dashed line indicates a neutral starting point of 0.5 for reference.

In both experiments, participants had to indicate as quickly as possible upon stimulus delivery whether they had received a low-intensity or high-intensity stimulation. Decision accuracy and response time were recorded as outcome parameters (Figure 1A–D). Using a hierarchical diffusion model [5], we compared the influence of the cues on drift rate (indicative of altered sensory processing) and on the starting point of the decision-making process (indicative of altered perceptual decision-making) by fitting a model that allowed for an influence of cue information on drift rate and starting point. Further free parameters of the model were non-decision time and boundary separation (see Supplemental Information for details). For each of the four parameters, Bayesian paired contrast tests were applied to test for effects of cue condition (Experiment 1: ‘80/20’, ‘or ‘50/50’; Experiment 2: ‘80/20’, ‘20/80’ or ‘50/50’) and stimulation intensity (i.e. high or low) and their interactions.

In both experiments, only the starting point showed a main effect of cue (for details see Supplemental Information), indicating that prior information biases perceptual decision-making (Figure 1E,F). In Experiment 1, participants showed a shift in starting point towards high intensity pain in the ‘80/20’ condition. In Experiment 2, the starting point was shifted towards high pain in the ‘80/20’ condition and towards low pain in the ‘20/80’ condition. The degree of shift in starting point away from the neutral starting point did not differ between the two conditions.

Both datasets also showed changes in drift rate (indicating altered sensory processing), which were, however, more closely related to the stimulation intensity than the cue condition. In Experiment 1, low-intensity stimuli yielded higher drift rates than high-intensity stimuli, irrespective of the cue condition. In Experiment 2, the drift rate in the ‘20/80’ condition was significantly increased if high-intensity stimuli were applied, indicating a ‘pop-out’ effect of unexpectedly high stimuli. None of the remaining comparisons, including those for non-decision time or boundary separation, reached significance.

The observation that prior information affects the perception of pain is not novel. There is ample evidence showing that pain can be amplified through negative expectations and reduced through expectations of pain relief [6]. However, neural mechanisms underlying these changes are still unclear. A prevalent explanation based on related studies using expectancy manipulations assumes that prior information changes the signal level in brain regions involved in processing sensory-discriminative aspects of pain. However, changes in pain perception can also be reflected in brain regions related to cognitive-affective processing [7]. Activation changes in sensory-discriminative brain regions are nevertheless considered the gold standard when proving genuine changes in pain perception as opposed to report bias.

Our data suggest that cognitive pain modulation can also be rooted in altered perceptual decision-making. Over recent years, the conceptualization of perception as an inferential process has critically changed our understanding of perception–cognition interactions. The basic tenet of this account is that incoming sensory information is not analyzed *de novo* but interpreted based on prior information. As a consequence, incoming information is more likely to be interpreted in accordance with the more likely percept. To date, the effect of prior information on perceptual decisions has mainly been studied in basic visual processing [8–10]. Our data critically extend these findings by showing that biased perceptual decision-making is pivotal to the modulation of pain, one of the most common and costly health care problems worldwide.

Our findings have several far-reaching implications. First, they challenge the current emphasis of neuroimaging studies investigating cognitive pain modulation on the search for changes in brain regions related to sensory-discriminative processing as too narrow. Research outside the pain domain has linked altered perceptual decision-making to activation changes in the anterior cingulate cortex (ACC) and the dorsolateral prefrontal cortex (DLPFC) [8], which have also been implicated in cognitive pain modulation [7]. Future studies have to identify neural processes underlying biased perceptual decision-making and probe their utility as objective indicators of pain modulation. Note that a bias in perceptual decision-making as observed here is not to be equated with report bias in which the report is decoupled from the perceptual process. Second, future studies have to specify the relative influence of processes such expectations, attention, uncertainty and feedback-driven learning that may underlie or mediate the effects of prior probability information and the generalisability of our findings for other types of peripheral input and perceptual experiences. Finally, it needs to be explored how our findings relate to previous studies in which the same stimulation intensity and probability was used in all conditions [1,2]. Modern conceptions of perception have begun to embrace evidence on cognitive influences onto perception. Our data strongly encourage this perspective to allow for a more comprehensive view on perception in general and clinical challenges such as pain in particular.

## Supplemental Information

Document S1. Supplemental Experimental Procedures, Two tables and One figure
