# Supplementary material for: Influence of prior information on pain involves biased perceptual decision-making
Source: Curr Biol. 2014 Aug 4;24(15):R679–81. doi: 10.1016/j.cub.2014.06.022 (PMC4123161; doi:10.1016/j.cub.2014.06.022)
Supplement: Document S1. Supplemental Experimental Procedures, Two tables and One figure [file mmc1.pdf]

Supplemental Information:

Influence of prior information on pain involves biased perceptual decision-making

Katja Wiech, Joachim Vandekerckhove, Jonas Zaman, Francis Tuerlinckx, Johan W.S. Vlaeyen, Irene Tracey

Supplemental Results

Experiment 1

Decision accuracies. As expected, accuracy rates for high intensity pain were higher in the '80/20' than in the '50/50' condition ($t(33)= 4.19$; $p< 0.001$; Figure 1A). Furthermore, accuracy rates for trials in which low intensity stimuli had been applied were higher in the '50/50' condition compared to the '80/20' condition ($t(33)= -3.28$; $p= 0.002$). A significant difference in accuracy was also found for the comparison of high and low intensity stimuli in the '50/50' condition ($t(33)= 4.47$; $p< 0.001$) with higher accuracies for the low intensity stimuli.

Response times (RTs). Fig. 1C shows RTs separately for correct and incorrect responses. In line with our finding of higher accuracies for the correctly cued high-intensity stimulation, participants were also faster in indicating their correct response when high-intensity stimuli had been preceded by the '80/20' cue than by the '50/50' cue ($t(33)= -4.31$; $p< 0.001$). Response times for the correct categorization of the low intensity stimuli, however, did not differ between both conditions ($t(33)= 0.97$; $p= 0.338$). Likewise, we found no significant difference in response time for the correct categorization of high and low intensity stimuli in the '50/50' condition ($t(33)= -1.24$; $p= 0.223$). Turning to the incorrect responses, incorrect categorizations of high intensity stimuli did not differ between the two cue conditions ($t(21)= 1.84$; $p= 0.079$). Likewise the RT difference between incorrect responses to low pain stimuli following the '80/20' cue and the '50/50' cue ($t(12)= 2.14$; $p= 0.053$) and the incorrect responses to the '50/50' cue for low and high intensity stimulation ($t(14)= 2.13$; $p= 0.052$) did not reach significance. However, in line with a decision-making bias, incorrect responses to the '80/20 cue' (i.e., categorization of high pain stimuli as low pain stimuli) were characterized by slower

RTs compared to correct responses to the same cue (i.e., categorization of high pain stimuli as high pain stimuli).

Experiment 2

Decision accuracies. Analysis of mean decision accuracies indicated that cue condition, ($F(1.94,40.81)= 3.48$, $p= 0.042$), but not stimulation intensity ($p> 0.05$) influenced the correct categorisation of painful stimuli as being either low-intensity or high-intensity (see Figure 1B). Post-hoc t-tests (corrected for multiple comparisons) revealed that accuracies in the 50/50 condition were generally higher than in the 20/80 condition ($p= 0.012$).

Furthermore, cue condition and stimulation intensity interacted to influence decision accuracies ($F(1.70, 35.61)= 5.26$, $p= 0.013$). To explore the nature of this interaction, we examined the influence of cue on decision accuracies following high-intensity and low-intensity pain separately. Bonferroni-corrected pairwise comparisons for the high intensity stimulation conditions did not reveal any significant effect ($p> 0.05$). In low intensity trials, participants made significantly more accurate decisions with the 50/50 cue than with the 80/20 cue ($t(21)= 2.80$, $p= 0.033$) whereas differences between the 80/20 and 20/80 condition and between the 20/80 and 50/50 cues were not significant (both $p> 0.05$). Last, accuracies for high-intensity trials and low-intensity trials in the '50/50' condition did not differ ($p > 0.05$).

Response times (RTs). Analysis of mean RTs of correctly classified trials revealed that condition and stimulation intensity alone did not influence RTs ($p> 0.05$ for both main effects) but showed a significant interaction ($F(2,23.32)= 33.23$; $p< 0.001$; Figure 1D). Bonferroni-corrected pairwise comparisons of conditions with the same stimulation intensity showed that RTs in the

‘80/20, high-intensity stimulation’ condition were significiantly shorter than in the ‘50/50’ ($t(20)= 4.84$) and the ‘20/80’ trials in which a high-intensity stimulus was applied ($t(20)= 5.07$; both $p< 0.001$). Furthermore, RTs of the ‘50/50’ condition were shorter than of the ‘20/80’ condition ($t(20)= 3.04$; $p= 0.018$). In the low intensity stimulation trials, the ‘20/80’ condition showed significantly shorter RTs than the ‘80/20’ condition ($t(20)= 5.69$) and the ‘50/50’ condition ($t(20)= 6.48$; both $p< 0.001$). The comparison between the ‘80/20’ and the ‘50/50’ condition did not reach significance. In incorrectly classified trials, both main effects (i.e., cue conditions and stimulation intensity) and their interaction were not significant.

As in Experiment 1, we also directly compared correct and incorrect responses of trials in which either high or low intensity stimulation was more likely (i.e., in the ‘80/20’ and the ‘20/80/ condition). These analyses revealed significantly longer RTs in incorrect than correct trials in the ‘80/20, high intensity stimulation’ trials ($t(11)= 3.26$, $p= 0.32$) and the ‘20/80, low intensity stimulation’ trials ($t(10)= 3.48$, $p= 0.024$).

Diffusion model.

Experiment 1

Change in starting point (indicative of perceptual decision-making bias). As shown in Figure 1E (left), the starting point in the ‘80/20’ condition, $\beta_{80:20}$, was significantly shifted towards high pain (upper boundary) compared to the ‘50/50’ condition ($Pr(\beta_{80:20} \leq \beta_{50:50}) < 0.0001$).[1]

[1] Here and throughout, Pr(X) indicates the posterior probability that a proposition X is true, given the data at hand. If this probability is very low, the complement of X is inferred. The four parameters of the model are starting point β, drift rate δ, boundary separation α, and non-decision time τ. Subscripts refer to conditions or averages over conditions, as appropriate. See Table S1 and Table S2 for exact formulas for all contrasts.

Drift rate (indicative of altered sensory processing). The drift rate was significantly increased in the low-intensity trials compared to the high-intensity trials across both cue conditions (main effect of stimulation intensity; $Pr(\delta_{low} \leq \delta_{high}) < 0.001$). In contrast, the main effect of cue condition and the interaction between both factors did not reach significance.

Non-decision time and boundary separation. Neither of the effects on non-decision time or boundary separation reached statistical significance (see Table S1 for details on all parameters).

Experiment 2

Change in starting point (indicative of perceptual decision-making bias). In Experiment 2 in which an additional third condition tested for the effects of prior information emphasizing low intensity stimulation, the starting point in the '20/80' condition was shifted towards low pain (lower boundary) relative to the '50/50' condition ($Pr(\beta_{50:50} \leq \beta_{20:80}) < 0.01$) and the '80/20' condition ($Pr(\beta_{80:20} \leq \beta_{20:80}) < 0.01$). The starting point in the '80/20' condition was clearly shifted towards high-intensity pain (see Figure 1F, left). The difference between the '80/20' condition and the '50/50' condition was less pronounced ($Pr(\beta_{80:20} \leq \beta_{50:50}) \approx .04$).

Drift rate (indicative of altered sensory processing). Pairwise comparisons testing for an influence of cue condition and stimulation intensity on drift rates revealed a difference in drift rate between '20/80' trials in which a high-intensity stimulation was applied and those in which the '20/80' cue was followed by the low-intensity stimulation ($Pr(\delta_{high,20:80} \leq \delta_{low,20:80}) \approx 0.02$)). As shown in Figure 1F (right), the drift rate was higher when the '20/80' cue was unexpectedly followed by a high-intensity stimulation compared to the expected low-intensity stimulation.

Non-decision time and boundary separation. Neither of the effects on non-decision time or boundary separation revealed effects (see Table S2 for details on all parameters).

Supplemental Experimental Procedures

Subjects. 34 right-handed healthy volunteers (23 female; mean age: 23.4 years) participated in Experiment 1 and 22 right-handed healthy volunteers (11 female; mean age: 26 years) took part in Experiment 2. Participants displayed normal pain thresholds at the site of stimulus application and had no history of neurological or psychiatric disease or chronic pain. All subjects participated having given full informed consent. The study was approved by the local Research Ethics committee.

Experimental design. In a probabilistic cueing paradigm, participants were presented with one of two (Experiment 1) or three (Experiment 2) visual cues in each trial. One cue signaled the subsequent application of a high intensity noxious electrical stimulus with a probability of 80% and of a low intensity stimulus with a probability of 20% ('80/20' condition). The second cue signaled a prior probability of 50% for both high and low intensity stimuli ('50/50' condition). In Experiment 2, a third cue signaled the delivery of a high intensity stimulus in 20% of the trials and of a low-intensity stimulus in 80% of the trials. The visual cues (the white outline of a square, triangle or circle) were randomly assigned to the conditions across subjects. Three seconds after the onset of the visual cue, the noxious stimulation was applied to the dorsum of the left hand. Participants were instructed to indicate as quickly as possible (without compromising on accuracy) whether they had received a low-intensity or high-intensity stimulation by pressing one of two buttons with their right hand. The decision accuracy and response time (i.e., time between delivery of the noxious stimulus and button press) were recorded as outcome parameters. No feedback was provided regarding the correctness of the response. Between consecutive trials a fixation cross was presented with a variable duration of

three, five or seven seconds. In Experiment 2, participants also provided ratings of the average intensity of pain and the average amount of anxiety experienced with each of the three cues at the end of each of the four blocks (data not shown here).

The experiment consisted of four blocks of 40 trials (Experiment 1) or 42 trials (Experiment 2) each. The order of conditions was pseudo-randomized in each block, with a maximum of two consecutive repetitions of the same condition. In Experiment 1, all participants underwent a practice run of 16 trials (eight 80/20 and eight 50/50 trials) before the actual experiment. In Experiment 2, they had to complete 24 practice trials (eight '80/20' trials, eight '50/50' trials and eight '20/80' trials).

Electrical stimulation. Prior to the practice run, individual stimulation levels for high and low pain were determined using the Method of Limits approach [S1]. The stimuli were applied to the back of the left hand using two commercial electric stimulation devices (Constant Current Stimulator, model DS7A; Digitimer©, Hertfordshire, UK) delivering a train of 1ms monopolar square waveform pulses via a concentric silver chloride electrode. Intensities rated as 2 on a Visual Analogue Scale ranging from 0 (= no pain) to 10 (= unbearable pain) were used for low pain. Intensities rated as VAS 6 (Experiment 1) or VAS 8 (Experiment 2) were used for high pain. In Experiment 1, the average stimulation intensity was 1.43 mA (SD= 0.94) for low pain stimuli and 4.37 mA (SD= 3.37) for high pain stimuli with a significant difference between both stimulation levels ($t(33)= -6.55$; $p< 0.001$). In Experiment 2, a mean stimulation intensity of 2.01 mA (SD= 1.54) was applied for low-intensity stimuli. The average intensity for high-intensity stimuli was 8.20 mA (SD= 4.15) which was significantly different from the low-intensity stimulation ($t(21) = 5.02$; $p< 0.001$). Prior to each block the participants' pain threshold was

recalibrated if necessary to ensure constant pain levels throughout the experiment. To this end, a series of stimuli of both stimulation intensities (i.e., current level that had been used to induce low and high intensity pain in the previous block) was applied and participants provided a verbal intensity rating for each stimulus. In case the rating deviated from the intended rating in more than two consecutive trials, the intensity was adjusted accordingly.

Experimental protocol. Upon arrival, participants were provided with a participant information sheet, were introduced to the experiment, familiarized with the equipment and they were given the possibility to ask questions. After they had provided informed consent, participants filled in the questionnaires on pain-related psychological measures (data not shown here). Subsequently, participants underwent the calibration procedure to determine the stimulation intensities required for the low level and the high level stimulation. Prior to the actual experiment, all participants completed a practice run (see above). Experiment 1 comprised four blocks of 40 trials each (20 trials of both conditions in a randomized order). Experiment 2 consisted of four blocks of 42 trials each (14 trials of each of the three conditions in a randomized order).

In order to test whether the low-intensity and the high-intensity stimulation were sufficiently different to allow for differential learning, participants performed a discrimination test prior to the practice run. In this stimulation discrimination test, ten low-intensity stimuli and ten high-intensity stimuli, which were adjusted to the individual stimulation level determined during the preceding calibration procedure were applied in a randomized order. Participants had to verbally categorize each stimulus as either a low-intensity or a high-intensity stimulus. If participants were able to classify at least 80% of the trials correctly, the actual experiment commenced. Categorization accuracies below 80% resulted in re-calibration and a second

practice run, which was successfully performed by all participants who required more practice. Furthermore, participants underwent a short test in which their understanding of the contingencies between the visual cues and the subsequent stimulation was probed. All cues were presented ten times in a randomized order and participants had to verbally indicate whether the cue signaled a high-intensity stimulation in 80%, 50% or 20% of the trials. Note that participants were explicitly informed about the contingencies prior to the performance test. Only if participants were able to categorize at least 80% of the trials correctly, the actual experiment was started. All participants were able to categorize the cues correctly at the first practice run.

Data analysis. All trials with a response time of two standard deviations above or below the group mean response time were excluded from the analysis (Experiment 1: mean exclusion rate: 3.4%; SD= 6.1; Experiment 2: mean exclusion rate: 4.3%; SD= 3.1). To characterize the effect of the cues on the decision, we first calculated the average response times (RT) for correct responses and accuracy rate (in % correct) separately for each conditions across the group. In Experiment 1, differences in RTs and accuracies between conditions were investigated for the following three comparisons using pairwise t-tests (adjusted for multiple comparisons using Bonferroni correction): (1) high $\text{pain}_{(80\%)}$ vs. high $\text{pain}_{(50\%)}$, (2) high $\text{pain}_{(50\%)}$ vs. low $\text{pain}_{(50\%),}$ (3) low $\text{pain}_{(20\%)}$ vs. low $\text{pain}_{(50\%)}$. In Experiment 2, we performed a repeated-measures ANOVA with the within-subject factors CUE CONDITION (i.e., ‘80/20’ cue, ‘50/50’ cue and ‘20/80’ cue) and STIMULATION INTENSITY (i.e., low-intensity and high-intensity stimulation) with post-hoc t-tests (corrected for multiple comparisons) for both, RTs and accuracies.

Model fitting. We estimated parameters of the hierarchical diffusion model (HDM) using the procedure, methods, and software described in Vandekerckhove et al. [S2] and Wabersich & Vandekerckhove [S3]. In short, the model is based on the drift diffusion process [S5,S6] that is assumed to underlie binary perceptual decisions. In this framework of sequential sampling models [S6], perception is understood as a statistical inference process in which sensory evidence is accumulated over time and the decision is made as soon as the upper or lower boundary is reached. Which boundary is reached determines which response is given. The most widely used model of the class of sequential sampling models is the diffusion model [S7] which is a computational representation of the decision process and makes particular predictions for choice response probabilities and response times. The decision process is characterized by a number of parameters, including the mean starting point β, the speed at which evidence is accumulated (i.e., the drift rate, δ), the boundary separation a that indicates the evidence required to make a response and the non-decision time τ that indicates the time used for everything except making a decision (i.e., encoding the stimulus and physically executing the response).

For two-choice decision-making we investigated here (i.e., the choice between high versus low intensity stimulation), there is a boundary for the correct response (responding "high"/"low" when the stimulus was high/low) and a boundary of an error response (responding "high"/"low" when the stimulus was low/high). The rate of information accumulation is related to the amount of information present in the stimulus. At the presentation of the stimulus, the information accumulation process starts in between the two boundaries. If a certain percept (e.g., high-intensity pain) is expected, the starting point in this inferential process will be closer to the corresponding correct boundary (e.g., boundary for high-intensity pain) and thereby

favor the expected percept. For instance, the cue that signals a prior probability of 80% for a high-intensity stimulation could shift the starting point towards the ‘high intensity’ decision boundary whereas the ~~non-informative~~ 50% probability cue can be expected to have no effect on the starting point. In the ‘~~informative (i.e., 80%)~~80/20’ trials compared to the ‘~~non-informative (50%)~~50/50’ trials, the shift in starting point is expected to lead to shorter response times during correct decisions, as well as a higher decision accuracy.

A bias in drift rate, in contrast, is characterized by accelerated evidence accumulation. Like the bias in starting point, it is associated with higher decision accuracies and shorter response times during correct decisions in the ‘80/20’ relative to ‘50/50’ condition trials. Note, however, that both types of bias differ in their effect on response times in error trials. While a bias in starting point leads to longer response times when validly cued high intensity stimuli are erroneously classified as low intensity stimulation (relative to errors in the ‘50/50’ condition), a bias in drift rate is characterized by shorter response times in these trials. By jointly considering response times and decision accuracies, the drift diffusion model can differentiate between both types of bias.

The classical drift diffusion model has proven useful in a variety of contexts. It does, however, not take into account the between-subject variability and is therefore less suitable for explaining inter-individual differences. In addition, studies in pain perception often administer only a modest number of trials (e.g., a few tens of trials rather than thousands as is common in standard psychophysical experiments). For such data structures, it is more advantageous to use hierarchical models so that information from different participants can be pooled [S8]. Therefore, a recently developed statistical implementation of the drift diffusion model, the

hierarchical diffusion model (HDM; [S2]) was applied. The HDM allows for the modeling of inter-individual differences by considering differences in parameter values between participants as random effects in the statistical sense.

Here, we fit the HDM in a Bayesian statistical framework, including both correct and incorrect trials. With the hierarchical model, we make the implied assumption that participants were randomly drawn from a larger population. The parameters to be estimated were the starting point (β), the drift rate (δ), the boundary separation (α), and the non-decision time (τ). The drift rate and non-decision time parameters were allowed to differ between all conditions of the experiment (four conditions in Experiment 1, six conditions in Experiment 2), while the boundary separation and starting point were allowed to differ as a function of task instruction only (two levels in Experiment 1, three levels in Experiment 2). Since these two parameters are by definition determined before the stimulus is presented and the information accumulation process starts, they cannot be affected by properties of the stimulus such as intensity. As part of the Bayesian analysis, we defined prior distributions for each parameter that reflect our knowledge about the parameter prior to observing the data. We defined uniform priors (i.e., uninformative prior distributions that did not favor one value over another) within a reasonable range for each parameter (ranges informed by the review data in [S9]; the results presented here were not sensitive to small changes in prior specification such as replacing a uniform distribution with a normal distribution with large variance). We then base our statistical inferences on the *posterior distributions* of the parameters because they represent our state of knowledge about the parameters after having taken into account the data. We summarize these posterior distributions by their means (i.e., leading to expected a posteriori or EAP estimates) and perform statistical inference by computing tail areas of contrast parameters. For

example, to test whether the decision bias parameter β is closer towards the upper boundary in the '50/50' condition compared to the '20/80' condition, we evaluate the proposition $\beta_{20:80} \leq \beta_{50:50}$, and so compute the posterior probability that $(\beta_{20:80} - \beta_{50:50}) \leq 0$. If this probability is high (greater than 97.5%), we infer that $\beta_{20:80} \leq \beta_{50:50}$. If it is low (less than 2.5%), we infer that $\beta_{20:80} > \beta_{50:50}$. If it is intermediate, we suspend judgment. Tail areas of contrast posteriors act as a Bayesian analogue to the classical *p*-value associated with a one-tailed test. Tables S1 and S2 contain all the planned contrasts expressed in terms of model parameters.

Table S1.

Summary statistics of the posterior distributions of all planned contrasts (Experiment 1).

Parameter	*Effect*	*Contrast (x)*	*Mean*	*SD*	*p(x<0)*
**α**	cue 50:50 - 80:20	$(\alpha_{50:50,\ high} - \alpha_{80:20,\ high} + \alpha_{50:50,\ low} - \alpha_{80:20,\ low})$ / 2;	-0.016	0.063	0.6002
**β**	cue 50:50 - 80:20	$(\beta_{50:50,\ high} - \beta_{80:20,\ high} + \beta_{50:50,\ low} - \beta_{80:20,\ low})$ / 2;	-0.160	0.038	<u>1.0000</u>
**τ**	intensity high - low	$(\tau_{80:20,\ high} + \tau_{50:50,\ high} - \tau_{80:20,\ low} - \tau_{50:50,\ low})$ / 2;	0.023	0.015	0.0595
**τ**	cue 50:50 - 80:20	$(\tau_{50:50,\ high} - \tau_{80:20,\ high} + \tau_{50:50,\ low} - \tau_{80:20,\ low})$ / 2;	-0.001	0.015	0.5267
**τ**	interaction 50:50 - 80:20	$(\tau_{50:50,\ high} - \tau_{80:20,\ high} - \tau_{50:50,\ low} + \tau_{80:20,\ low})$;	0.044	0.030	0.0684
**δ**	intensity high - low	$(\delta_{80:20,\ high} + \delta_{50:50,\ high} - \delta_{80:20,\ low} - \delta_{50:50,\ low})$ / 2;	1.452	0.312	<u>0.0000</u>
**δ**	cue 50:50 - 80:20	$(\delta_{50:50,\ high} - \delta_{80:20,\ high} + \delta_{50:50,\ low} - \delta_{80:20,\ low})$ / 2;	-0.157	0.301	0.7005
**δ**	interaction 50:50 - 80:20	$(\delta_{50:50,\ high} - \delta_{80:20,\ high} - \delta_{50:50,\ low} + \delta_{80:20,\ low})$;	-0.591	0.618	0.8317

Table S2.

Summary statistics of the posterior distributions of all planned contrasts (Experiment 2).

Parameter	***Effect***	***Contrast (x)***	***Mean***	***SD***	***p(x<0)***
**α**	cue 50:50 - 80:20	$(\alpha_{50:50,\ high} - \alpha_{80:20,\ high} + \alpha_{50:50,\ low} - \alpha_{80:20,\ low})\ /\ 2$	0.024	0.119	0.4266
**α**	cue 20:80 - 50:50	$(\alpha_{20:80,\ high} - \alpha_{50:50,\ high} + \alpha_{20:80,\ low} - \alpha_{50:50,\ low})\ /\ 2$	0.045	0.123	0.3348
**α**	cue 20:80 - 80:20	$(\alpha_{20:80,\ high} - \alpha_{80:20,\ high} + \alpha_{20:80,\ low} - \alpha_{80:20,\ low})\ /\ 2$	0.069	0.113	0.2706
**β**	cue 50:50 - 80:20	$(\beta_{50:50,\ high} - \beta_{80:20,\ high} + \beta_{50:50,\ low} - \beta_{80:20,\ low})\ /\ 2$	-0.09	0.048	0.9616
**β**	cue 20:80 - 50:50	$(\beta_{20:80,\ high} - \beta_{50:50,\ high} + \beta_{20:80,\ low} - \beta_{50:50,\ low})\ /\ 2$	-0.182	0.05	0.9953
**β**	cue 20:80 - 80:20	$(\beta_{20:80,\ high} - \beta_{80:20,\ high} + \beta_{20:80,\ low} - \beta_{80:20,\ low})\ /\ 2$	-0.272	0.061	0.9962
**β**	distance from 50:50	$(\beta_{20:80,\ high} - 2\beta_{50:50,\ high} + \beta_{80:20,\ high} + \beta_{20:80,\ low} - 2\beta_{50:50,\ low} + \beta_{80:20,\ low})/2$	-0.093	0.076	0.8887
**β**	distance from β = 0.5	$((\beta_{20:80,\ high} - 0.5) - (0.5 - \beta_{80:20,\ high}) + (\beta_{20:80,\ low} - 0.5) - (0.5 - \beta_{80:20,\ low}))/2$	-0.013	0.042	0.6205
**τ**	intensity high - low	$(\tau_{80:20,\ high} + \tau_{50:50,\ high} + \tau_{20:80,\ high} - \tau_{80:20,\ low} - \tau_{50:50,\ low} - \tau_{20:80,\ low})\ /\ 3$	0.017	0.018	0.1729
**τ**	cue 50:50 - 80:20	$(\tau_{50:50,\ high} - \tau_{80:20,\ high} + \tau_{50:50,\ low} - \tau_{80:20,\ low})\ /\ 2$	0.028	0.022	0.1011
**τ**	cue 20:80 - 50:50	$(\tau_{20:80,\ high} - \tau_{50:50,\ high} + \tau_{20:80,\ low} - \tau_{50:50,\ low})\ /\ 2$	-0.044	0.024	0.96
**τ**	cue 20:80 - 80:20	$(\tau_{20:80,\ high} - \tau_{80:20,\ high} + \tau_{20:80,\ low} - \tau_{80:20,\ low})\ /\ 2$	-0.015	0.021	0.7605
**τ**	interaction 50:50 - 80:20	$(\tau_{50:50,\ high} - \tau_{80:20,\ high} - \tau_{50:50,\ low} + \tau_{80:20,\ low})$	-0.036	0.045	0.7904
**τ**	interaction 20:80 - 50:50	$(\tau_{20:80,\ high} - \tau_{50:50,\ high} - \tau_{20:80,\ low} + \tau_{50:50,\ low})$	0.033	0.047	0.2261
**τ**	interaction 20:80 - 80:20	$(\tau_{20:80,\ high} - \tau_{80:20,\ high} - \tau_{20:80,\ low} + \tau_{80:20,\ low})$	-0.003	0.05	0.4995
**δ**	intensity high - low	$(\delta_{80:20,\ high} + \delta_{50:50,\ high} + \delta_{20:80,\ high} - \delta_{80:20,\ low} - \delta_{50:50,\ low} - \delta_{20:80,\ low})\ /\ 3$	-0.054	0.184	0.6149
**δ**	cue 50:50 - 80:20	$(\delta_{50:50,\ high} - \delta_{80:20,\ high} + \delta_{50:50,\ low} - \delta_{80:20,\ low})\ /\ 2$	0.119	0.205	0.2782
**δ**	cue 20:80 - 50:50	$(\delta_{20:80,\ high} - \delta_{50:50,\ high} + \delta_{20:80,\ low} - \delta_{50:50,\ low})\ /\ 2$	0.041	0.206	0.4201

**δ**	cue 20:80 - 80:20	$(\delta_{20:80,\ high} - \delta_{80:20,\ high} + \delta_{20:80,\ low} - \delta_{80:20,\ low}) / 2$	0.16	0.209	0.2211
**δ**	interaction 50:50 - 80:20	$(\delta_{50:50,\ high} - \delta_{80:20,\ high} - \delta_{50:50,\ low} + \delta_{80:20,\ low})$	-0.164	0.473	0.6444
**δ**	interaction 20:80 - 50:50	$(\delta_{20:80,\ high} - \delta_{50:50,\ high} - \delta_{20:80,\ low} + \delta_{50:50,\ low})$	-1.009	0.477	0.9795
**δ**	interaction 20:80 - 80:20	$(\delta_{20:80,\ high} - \delta_{80:20,\ high} - \delta_{20:80,\ low} + \delta_{80:20,\ low})$	-1.173	0.55	0.969
**δ**	pop-out 20:80	$\delta_{20:80,\ low} - \delta_{20:80,\ high}$	0.782	0.356	0.0200
**δ**	pop-out 50:50	$\delta_{50:50,\ low} - \delta_{50:50,\ high}$	-0.228	0.309	0.7713
**δ**	pop-out 80:20	$\delta_{80:20,\ low} - \delta_{80:20,\ high}$	-0.392	0.361	0.8704

Figure S1.

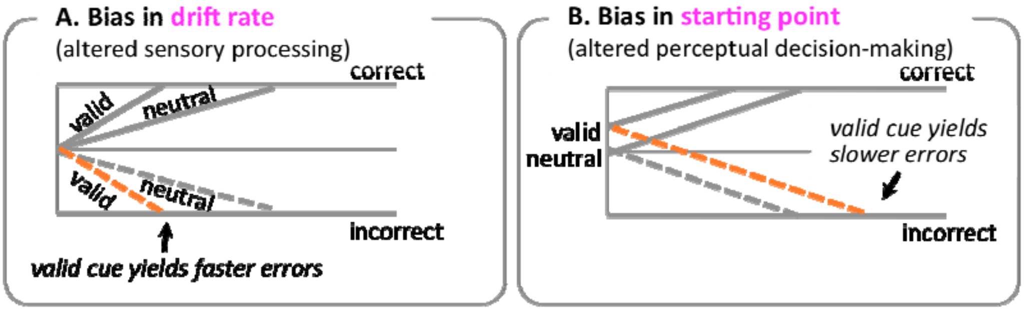

Biased sensory processing or altered perceptual decision-making? If a higher prior probability for high-intensity pain leads to a change in sensory processing (A), incorrect responses are characterized by shorter responses times (RTs) relative to RTs under changes in perceptual decision-making (B). Figure adapted from [S10].

Supplemental References

S1. Fruhstorfer, H., Lindblom, U., and Schmid, W. C. (1976). Method for quantitative estimation of thermal thresholds in patients. J. Neurol. Neurosurg. Psychiatry *39*, 1071.

S2. Vandekerckhove, J., Tuerlinckx, F., and Lee, M. D. (2011). Hierarchical diffusion models for two-choice response times. Psychol. Methods *16*, 44–62.

S3. Wabersich, D., and Vandekerckhove, J. (2014). Extending JAGS: A tutorial on adding custom distributions to JAGS (with a diffusion model example). Behav. Res. Methods *46*, 15–28.

S4. Laming, D. R. J. (1968). Information theory of choice-reaction times (London: Academic Press).

S5. Link, S. W., and Heath, R. A. (1975). A sequential theory of psychological discrimination. Psychometrika *40*, 77–105.

S6. Smith, P. L., and Ratcliff, R. (2004). Psychology and neurobiology of simple decisions. Trends Neurosci. *27*, 161–168.

S7. Ratcliff, R. (1978). A theory of memory retrieval. Psychol. Rev. *85*, 59–108.

S8. Snijders, T. A. B., and Bosker, R. (2011). Multilevel analysis: An introduction to basic and advanced multilevel modeling (London: Sage Publications Ltd.).

S9. Matzke, D., and Wagenmakers, E. J. (2009). Psychological interpretation of the ex-Gaussian and shifted Wald parameters: a diffusion model analysis. Psychon. Bull. Rev. *16*, 798–817.

S10. Mulder, M. J., Wagenmakers, E. J., Ratcliff, R., Boekel, W., and Forstmann, B. U. (2012). Bias in the brain: A diffusion model analysis of prior probability and potential payoff. J. Neurosci. *32*, 2335–2343.

APPENDIX

Here we provide additional information about the definition of the hierarchical diffusion model used in this publication.

The model was fit in JAGS using the jags-wiener plugin for the diffusion model. MATLAB was used to interface with JAGS. MATLAB code can be obtained from Joachim Vandekerckhove.

FULL DEFINITION OF THE HIERARCHICAL DIFFUSION MODEL: JAGS CODE

```
model {
    # Priors
    v.mu[1,1] ~ dunif(-5.00, 5.00)     # 80/20, high
    v.mu[1,2] ~ dunif(-5.00, 5.00)     # 80/20, low
    v.mu[2,1] ~ dunif(-5.00, 5.00)     # 50/50, high
    v.mu[2,2] ~ dunif(-5.00, 5.00)     # 50/50, low
    v.mu[3,1] ~ dunif(-5.00, 5.00)     # 20/80, high
    v.mu[3,2] ~ dunif(-5.00, 5.00)     # 20/80, low

    b.mu[1,1]   ~ dunif( 0.01, 0.99)   # 80/20, high
    b.mu[2,1]   ~ dunif( 0.01, 0.99)   # 50/50, high
    b.mu[3,1]   ~ dunif( 0.01, 0.99)   # 20/80, high
    b.mu[1,2]   <- b.mu[1,1]           # 80/20, low
    b.mu[2,2]   <- b.mu[2,1]           # 50/50, low
    b.mu[3,2]   <- b.mu[3,1]           # 20/80, low

    Ter.mu[1,1] ~ dunif( 0.05, 1.00)   # 80/20, high
    Ter.mu[1,2] ~ dunif( 0.05, 1.00)   # 80/20, low
    Ter.mu[2,1] ~ dunif( 0.05, 1.00)   # 50/50, high
    Ter.mu[2,2] ~ dunif( 0.05, 1.00)   # 50/50, low
    Ter.mu[3,1] ~ dunif( 0.05, 1.00)   # 20/80, high
    Ter.mu[3,2] ~ dunif( 0.05, 1.00)   # 20/80, low

    a.mu[1,1]   ~ dunif( 0.40, 3.00)   # 80/20, high
    a.mu[2,1]   ~ dunif( 0.40, 3.00)   # 50/50, high
    a.mu[3,1]   ~ dunif( 0.40, 3.00)   # 20/80, high
    a.mu[1,2]   <- a.mu[1,1]           # 80/20, low
    a.mu[2,2]   <- a.mu[2,1]           # 50/50, low
    a.mu[3,2]   <- a.mu[3,1]           # 20/80, low

    a.sd    ~ dunif( 0.001, 0.50)  # population SD for a
    a.tau    <- pow(a.sd, -2)
    b.sd    ~ dunif( 0.001, 0.25)  # population SD for b
    b.tau    <- pow(b.sd, -2)
    Ter.sd ~ dunif( 0.001, 0.30)  # population SD for Ter
    Ter.tau <- pow(Ter.sd, -2)
    v.sd    ~ dunif( 0.001, 3.00)  # population SD for v
    v.tau    <- pow(v.sd, -2)

    st      ~ dunif(0.001, 0.30)  # inter-trial SD for Ter
    pt       <- pow(st, -2)

    # Hierarchical level
    for(p in 1:P) {  # persons
        for (i in 1:I) {  # intensities
           for (t in 1:C) {  # cues
                b[t,i,p]   ~ dnorm(b.mu[t,i]  , b.tau  )T( 0.01, 0.99)
                a[t,i,p]   ~ dnorm(a.mu[t,i]  , a.tau  )T( 0.10, 3.50)
```

```
              Ter[t,i,p] ~ dnorm(Ter.mu[t,i], Ter.tau)T( 0.00,     )
              v[t,i,p]   ~ dnorm(v.mu[t,i]  , v.tau  )T(-7.50, 7.50)
          }
      }
  }

  # Likelihood
  for (i in 1:N)
  {
      ai[i] <- a[shape[i], intensity[i], person[i]]
      bi[i] <- b[shape[i], intensity[i], person[i]]
      vi[i] <- v[shape[i], intensity[i], person[i]]
      ti[i] ~ dnorm(Ter[shape[i], intensity[i], person[i]], pt)
      y[i] ~ dwiener(ai[i], ti[i], bi[i], vi[i])  # likelihood
  }
}
```
